# Pediatric acute myeloid leukemia with t(8;21) and *KIT* mutation treatment with avapritinib post-stem cell transplantation: a report of four cases

**DOI:** 10.1007/s00277-024-05810-z

**Published:** 2024-05-28

**Authors:** Qingwei Wang, Yixin Hu, Li Gao, Senlin Zhang, Jun Lu, Bohan Li, Jie Li, Yanhua Yao, Shengqin Cheng, Peifang Xiao, Shaoyan Hu

**Affiliations:** Department of Hematology and Oncology, Children’s Hospital of Soochow University, No. 92, Zhongnan Street, Suzhou, 215000 China

**Keywords:** Acute myeloid leukemia, Avapritinib, *KIT* mutation, Pediatric, t(8;21)

## Abstract

Acute myeloid leukemia (AML) with t(8;21) (q22;q22), which forms *RUNX1::RUNX1T1* fusion gene, is classified as a favorable-risk group. However, the presence of mutations in *KIT* exon 17 results in an adverse prognosis in this group. Avapritinib, a novel tyrosine kinase inhibitor, was designed to target *KIT* mutation. We report a retrospective study of four pediatric patients with AML with t(8:21) and *KIT* exon 17 mutation who were treated with avapritinib, three of them failed to demethylate drugs and donor lymphocyte infusion targeting *RUNX1::RUNX1T1*-positivity after allogeneic hematopoietic stem cell transplantation (allo-HSCT). So far, all patients with *RUNX1::RUNX1T1* positivity had turned negative after 1, 9, 7, 2 months of avapritinib treatment. The common adverse effect of avapritinib is neutropenia, which is well-tolerated. This case series indicates that avapritinib may be effective and safe for preemptive treatment of children with AML with t(8;21) and *KIT* mutation after allo-HSCT, providing a treatment option for preventing relapse after allo-HSCT.

## Introduction

Core binding factor acute myeloid leukemia (CBF-AML) includes AML cases harboring *RUNX1::RUNX1T1* or *CBFβ::MYH11* fusion genes, which are classified as a favorable-risk group [1, 2]. However, 20–40% of adult patients with *de novo* CBF-AML [3–7] and 23% of children with CBF-AML [8] have *KIT* mutation with varied impacts on prognosis. Several studies have proposed that patients with AML with *RUNX1::RUNX1T1* as well as *KIT* mutation [9], particularly *KIT* exon 17 mutation, have worse clinical outcomes [10, 11]. Patients with high-risk features can benefit from allogeneic hematopoietic stem cell transplantation (allo-HSCT), but 20% of patients with AML with *RUNX1::RUNX1T1* relapse after that and the dynamics of the transcript levels can predict relapse in these patients [12, 13] .

Avapritinib, a novel tyrosine kinase inhibitor targeting *KIT/PDGFRA*, has been approved for treating gastrointestinal stromal tumors and systemic mastocytosis. It effectively treats minimal residual disease (MRD) in AML cases harboring *RUNX1::RUNX1T1* and *KIT* mutations after allo-HSCT [14]. However, clinical application in pediatric AML is lacking. Here, we report four children with AML with *RUNX1::RUNX1T1* and *KIT* mutations, who mostly were refractory to demethylation drugs (HMAs) and donor lymphocyte infusion (DLI), who were treated with avapritinib as a preemptive intervention after allo-HSCT.

## Methods

**Fig. 1:**
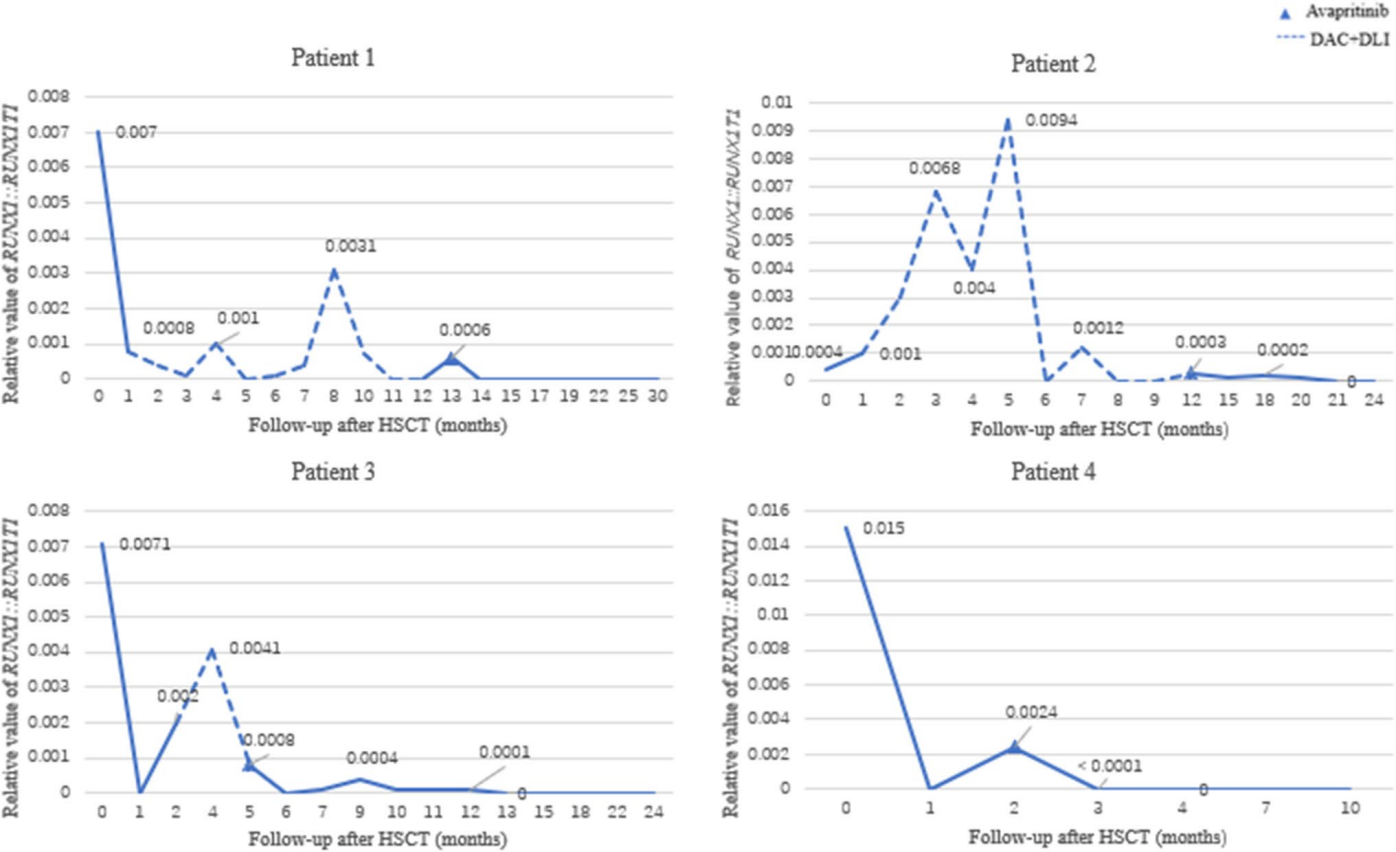
Relative value of *RUNX1::RUNX1T1* after allo-HSCT.  0 represents the last relative value of *RUNX1::RUNX1T1* before allo-HSCT. DLI, donor lymphocyte infusion; DAC, decitabine

The patient characteristics and treatment are summarized in Table 1. They were treated using the CALSIII-AML18 protocol, registered as ChiCTRl800015883 (the registration date: April 26, 2018), and it is is comparable that the result of long-term survival between standard-dose chemotherapy (SDC) low-dose chemotherapy (LDC) [15]. All four patients underwent HSCT with myeloablative conditioning regimens after achieving complete remission in morphology and the transplantation circumstances were shown in the Table 2. They underwent painless puncture for monitoring disease status dynamically. MRD was monitored by flow cytometry (FCM), the *RUNX1::RUNX1T1* (Fig. 1) transcript level was measured quantitatively by real-time quantitative reverse transcription polymerase chain reaction (RT-qPCR) and *KIT* mutation status was monitored by direct sequencing. Four patients were treated with avapritinib for *RUNX1::RUNX1T1*-positive AML in spite of complete remission in morphology, MRD by FCM and *KIT* mutation after allo-HSCT and three of them failed to HMAs and DLI. The starting dose of avapritinib was 50 mg/day and the administration would be adjusted if the patients experienced ≥ grade 3 adverse events (Table 3). The content and standards of adverse events were based on the Common Adverse Event Evaluation Standards (CTCAE) Version 5.0 [16]. The study protocol was approved by the Ethics Committee of Children’s Hospital of Soochow University, and informed consent was obtained from the parents of the patients. The data cut-off date for the primary analysis was January 2024.

**Table 1:** Baseline characteristics of *KIT*-mutant AML patients

NO	Age/sex	KIT mutation	Other mutations	Chemotherapy^a^	Time from diagnosis to first relapse (month)	BM status before HSCT (Morphology / FCM-MRD /Fusion gene (relative value) /KIT)	Pretreatment scheme^b^
1	12/F	*KIT*: D816V	KRAS、ASXL1	CALSIII-AML18 protocol (SDC ^c^)	none	2%/<0.1% / 0.0007/negative	CCNU + CLAG + DEX + BuCy
2	4/M	*KIT*: N822K、*KIT*: V560D	NRAS、FLT3	CALSIII-AML18 protocol (LDC ^d^)	none	1%/<0.1%/0.0004/negative	CCNU + CLAG + DEX + BuCy + ATG
3	12/M	*KIT*: D816Y	KDM6A	CALSIII-AML18 protocol (SDC)	none	1%/<0.1%/0.0071/negative	CCNU + CLAG + DEX + BuCy + ATG
4	11/M	*KIT*: N822K	none	CALSIII-AML18 protocol (SDC)	2	1%/6.01%/0.0150/NA	CCNU + CLAG + DEX + BuCy + ATG

*BM*, bone marrow; *HSCT*, hematopoietic stem cell transplantation; FCM-*MRD*, measurable residual disease monitored by flow cytometry; *NA*, not available. ^a^ All patients were treated by the protocol registered as ChiCTRl800015883. ^b^ All patients in this study received myeloablative conditioning regimens. ^c^ Standard-dose chemotherapy. ^d^ Low-dose cytarabine and mitoxantrone or omacetaxine mepesuccinate with concurrent granulocyte colony-stimulating factor (low-dose chemotherapy); *CCNU*, cyclonexyl-chloroethyl-nitrosourea; *CLAG*, Cladribine, Cytarabine, granulocyte colony-stimulating factor; *DEX*, Dexamethasone; *BuCy* Busulfan, Cyclophosphamide; *ATG* antithymocyte globulin

**Table 2:** Transplantation circumstances of *KIT*-mutant AML patients

NO	HLA-matched site	Cell doses of MNC (10^^8^ /kg)	Cell doses of CD34+ (10^^6^ /kg)	ANC engraftment (day)	PLT engraftment (day)	From HSCT to Molecular relapse (month)	DLI^a^ (times)	DAC^b^
1	10/10	9.85	5.50	12	11	1	9	10 mg/m^2^*3d /course *7 courses
2	10/10	6.74	11.91	11	11	1	4	10 mg/m^2^*5d /course *8 courses
3	5/10	6.03	8.30	13	13	2	1	10 mg/m^2^*5d /course*3 courses
4	5/10	5.80	10.17	12	13	2	none	none

*AML*, acute myeloid leukemia; *HLA*, human leukocyte antigen;* MNC*, mononuclear cell; *ANC*, absolute neutrophil count; *PLT*, platelet; *DLI*, donor lymphocyte infusions; *DAC*, decitabine; ^a^ CD3 + T cells were at least 0.5-1 × 10^7^ /kg once a month. ^b^10 mg/m^2^ for 3–5 days continuously in a month as a course

**Table:** Evaluation of treatment with avapritinib

NO	Usage	Response	Adverse events(grade)	Survival status	Follow-up since HSCT (months)
1	50 mg QOD	Molecular Negative after 1 month	neutropenia (4), thrombocytopenia (2), puffiness (1)	Alive	30
2	50 mg QD	Molecular Negative after 9 months	neutropenia (2), puffiness (1)	Alive	24
3	50 mg QOD	Molecular Negative after 7 months	neutropenia (3)	Alive	24
4	50 mg QD	Molecular Negative after 2 months	neutropenia (3)	Alive	10

*QOD*, once every other day; *QD*, once a day

### Case 1

A 12-year-old girl was admitted to our hospital with upper limb pain on March 19, 2021. A *RUNX1::RUNX1T1*-fusion gene and *KIT* D816V mutation were detected by RT-qPCR and next-generation sequencing, respectively. She was treated with the CALSIII-AML18 protocol (SDC), after which morphology normalized, MRD by FCM and *KIT* mutation had disappeared; however, *RUNX1::RUNX1T1*-positivity remained prior to allo-HSCT. To reduce relapse risk, she was transfused with human leukocyte antigen (HLA) 10/10 peripheral blood (PB) stem cells. *RUNX1::RUNX1T1*-positivity remained present in the first month after allo-HSCT. Decitabine (DAC) (10 mg/m^2^, 3 days/course, 7 courses) and DLI (9 cycles) were administered preemptively, and the fusion gene load was alleviated; however, negativity was not sustained and *RUNX1::RUNX1T1* progressed once we tried stopping DAC and DLI trentment. Although no severe graft-versus-host disease (GVHD) during this period, adverse events such as abnormal liver function (grade 3) and neutropenia (grade 4) occurred. To prevent the progression of the disease, she was given avapritinib monotherapy, *RUNX1::RUNX1T1*-negativity was achieved after 1 month and has been 16 months. During avapritinib monotherapy, neutropenia (grade 4), thrombocytopenia (grade 2), and puffiness (grade 1) occurred, which improved after adjustment of medication.

### Case 2

On August 23, 2021, a 4-year-old boy with t(8;21) and *KIT* N822K and *KIT* V560D mutations presented to the hospital with commissure distortion. The patient was treated with the CALSIII-AML18 protocol (LDC), with dasatinib during chemotherapy because of *KIT* mutation. He achieved a morphological complete response and *KIT*-negativity; however, *RUNX1::RUNX1T1* remained positive before allo-HSCT. He was transfused with unrelated donor HLA 10/10 PB stem cells. *RUNX1::RUNX1T1* also tested positive in the first month after allo-HSCT and showed a rising trend. DAC (10 mg/m^2^, 5 days/course, 8 courses) and DLI (4 cycles) did not maintain fusion gene-negativity and caused neutropenia (grade 4) in spite of without GVHD. The copy of transcript continued to decrease, turned negative after 9 months of avapritinib monotherapy and had stayed for 3 months. During the period, neutropenia (grade 2), puffiness (grade 1) occurred and those could improve after clinical observation.

### Case 3

The third patient with CBF-AML was a 12-year-old boy with *KIT* D816Y-mutated who was hospitalized for ecchymosis of the skin on June 9, 2021. He was treated using the CALSIII-AML18 protocol (SDC) and achieved the same bone marrow (BM) remission status as Cases 1 and 2, before transfusion with his brother’s HLA 5/10 PB stem cells. *RUNX1::RUNX1T1* fusion gene level was positive in the second month after allo-HSCT. DAC (10 mg/m^2^, 5 days/course, 3 courses) and DLI (once) failed to turn the fusion gene negative while caused grade II aGVHD and neutropenia (grade 3). After 7 months of avapritinib monotherapy, *RUNX1::RUNX1T1* gene tests turned negative and has kept for 11 months. Although it happened to neutropenia (grade 3), which also improved by adjustment of medication.

## Case 4

The fourth AML patient was an 11-year-old boy with *KIT* N822K and t(8;21) who visited the hospital with anemia and bleeding on April 6, 2022. He was primarily treatment refractory/resistant, and salvage therapy before allo-HSCT was suggested after a BM smear showed 6.01% MRD by FCM though CR in morphology. He was transfused with HLA 5/10 BM and PB stem cells. *RUNX1::RUNX1T1* tests were positive in the second month after allo-HSCT. Due to the first three patients did not respond quickly to DAC and DLI, the fourth patient was directly administered avapritinib monotherapy for preemptive intervention to observe the efficacy and exclude the intervention of DAC and DLI. After 2 months of preemptive treatment, the fusion gene turned negative and disease-free state has been maintained for 7 months. He also suffered neutropenia (grade 3) and improved after clinical observation.

## Discussion

*KIT* is composed of 21 exons and is located on chromosomal segment 4q11 [17]. Exon 8 and 17 mutations commonly occur in AML, particularly in exon 17, which are most strongly related to inferior prognosis in patients with *de novo* AML with t(8;21) [3, 7, 18], and allo-HSCT may improve prognosis in these patients [19]. Our four cases had AML with *RUNX1::RUNX1T1* and *KIT* exon 17 mutations and all patients achieved complete morphological remission before allo-HSCT; however, *RUNX1::RUNX1T1*-positivity was found within 60 days after allo-HSCT. Preemptive HMAs and DLI following allo-HSCT can prevent relapse in high-risk AML cases [20–22]. Three of our patients received DAC and DLI because of *RUNX1::RUNX1T1*-positivity after allo-HSCT, but fusion gene-negativity could not be sustained and case 3 occurred to grade II aGVHD. A previous study demonstrated that AML patients with *RUNX1::RUNX1T1* and *KIT* mutation in relapse after allo-HSCT show a rapid response to avapritinib [14, 23], 80% of such patients achieved the decrease of *RUNX1::RUNX1T1*-positivity transcript levels after 1 month of avapritinib treatment for MRD [14]. Our four patients achieved sustained fusion gene-negativity after 1, 9, 7, 2 months of avapritinib treatment and stayed complete remission until now. Because the establishment of graft-versus-leukemia effect of allo-HSCT and DAC + DLI can take some time, the complete remission in our patients cannot be attributed solely to avapritinib. However, none of the three patients maintained negative transcript more than a month during DAC and DLI while case 4 had an effective and quick performance to avapritinib preemptive monotherapy, which supported they could bebefit from avapritinib as preemptive therapy after HSCT.

The most common adverse event associated with avapritinib treatment is myelosuppression. In a study of patients with gastrointestinal stromal tumors [24], decreased hemoglobin, while in AML patients [14], thrombocytopenia was the most common hematological adverse event after avapritinib. This could be related to the heterogeneity of different diseases, avapritinib doses, and sample sizes. In our study, the most common adverse event was neutropenia, of which three of the four patients had grade 3–4. One patient developed grade 2 thrombocytopenia. Besides, two patients had grade 1 puffiness. All adverse events were tolerated and improved after clinical observation and medication adjustments compared to DAC and DLI. All the patients have not stopped avapritinib and survived with disease-free to the present date.

In previous studies [14, 23, 25], avapritinib doses mostly ranged from 50 to 200 mg/day and 50 mg/day as an initial dose was recommended to 2 children. In our study, 50 mg/day was also used as the initial dose, adjusted according to children’s tolerance, which could be a clinical reference for children’s medication. However, no study indicates the recommended duration of avapritinib as preemptive treatment for patients with AML after allo-HSCT. In addition, our sample size was too small to evaluate the clinical efficacy and safety of avapritinib. As a retrospective study, some bias may have existed in patient selection. Further studies and an observation period are required to address these issues. We have started a clinical trial (GMCAIII, registered as NCT06221683) in which the dose was based on these four patients used.

In conclusion, in our patients, avapritinib was well-tolerated and can be effective as a preemptive treatment of pediatric AML with *KIT* mutation after allo-HSCT.

## Abbreviations

AML: acute myeloid leukemia
MRD: measurable residual disease
allo-HSCT: allogeneic hematopoietic stem cell transplantation
CBF-AML: core binding factor acute myeloid leukemia
HMAs: demethylation drugs
DLI: donor lymphocyte infusion
PB: peripheral blood
BM: bone marrow
RT-qPCR: Real-time quantitative reverse transcription-polymerase chain reaction
DAC: decitabine
